# Exploring regression dilution bias using repeat measurements of 2858 variables in ≤49 000 UK Biobank participants

**DOI:** 10.1093/ije/dyad082

**Published:** 2023-06-19

**Authors:** Charlotte E Rutter, Louise A C Millard, Maria Carolina Borges, Deborah A Lawlor

**Affiliations:** MRC Integrative Epidemiology Unit, University of Bristol, Bristol, UK; Department of Medical Statistics, London School of Hygiene and Tropical Medicine (LSHTM), London, UK; MRC Integrative Epidemiology Unit, University of Bristol, Bristol, UK; Population Health Sciences, Bristol Medical School, University of Bristol, Bristol, UK; MRC Integrative Epidemiology Unit, University of Bristol, Bristol, UK; Population Health Sciences, Bristol Medical School, University of Bristol, Bristol, UK; MRC Integrative Epidemiology Unit, University of Bristol, Bristol, UK; Population Health Sciences, Bristol Medical School, University of Bristol, Bristol, UK; Bristol NIHR Biomedical Research Centre, University of Bristol, Bristol, UK

**Keywords:** Regression dilution bias, concordance, random measurement error, random variability, attenuation by errors, UK Biobank

## Abstract

**Background:**

Measurement error in exposures and confounders can bias exposure–outcome associations but is rarely considered. We aimed to assess random measurement error of all continuous variables in UK Biobank and explore approaches to mitigate its impact on exposure–outcome associations.

**Methods:**

Random measurement error was assessed using intraclass correlation coefficients (ICCs) for all continuous variables with repeat measures. Regression calibration was used to correct for random error in exposures and confounders, using the associations of red blood cell distribution width (RDW), C-reactive protein (CRP) and 25-hydroxyvitamin D [25(OH)D] with mortality as illustrative examples.

**Results:**

The 2858 continuous variables with repeat measures varied in sample size from 109 to 49 121. They fell into three groups: (i) baseline visit [529 variables; median (interquartile range) ICC = 0.64 (0.57, 0.83)]; (ii) online diet by 24-h recall [22 variables; 0.35 (0.30, 0.40)] and (iii) imaging measures [2307 variables; 0.85 (0.73, 0.94)]. Highest ICCs were for anthropometric and medical history measures, and lowest for dietary and heart magnetic resonance imaging.

The ICCs (95% confidence interval) for RDW, CRP and 25(OH)D were 0.52 (0.51, 0.53), 0.29 (0.27, 0.30) and 0.55 (0.54, 0.56), respectively. Higher RDW and levels of CRP were associated with higher risk of all-cause mortality, and higher concentration of 25(OH)D with lower risk. After correction for random measurement error in the main exposure, the associations all strengthened. Confounder correction did not influence estimates.

**Conclusions:**

Random measurement error varies widely and is often non-negligible. For UK Biobank we provide relevant statistics and adaptable code to help other researchers explore and correct for this.

Key Messages

- Random measurement error in the exposure and confounders can bias the association between exposure and outcome towards or away from the null.
- Some prospective studies, including UK Biobank, provide repeat measures of variables in a subsample for exploring bias due to random measurement error; these are rarely used.
- Our results demonstrate that measurement error is often non-negligible and may bias estimates.
- Agreement between repeated measures in UK Biobank varies by category of phenotype. Dietary and heart magnetic resonance imaging variables are least stable whereas anthropometric and medical history variables are most stable.
- We have provided intraclass correlation coefficients for 2858 continuous variables from UK Biobank and adaptable code to support researchers to correct for bias due to random error in exposures and confounders.

## Introduction

Measurement error is a widespread problem when estimating the association between two variables and can be random (where measurements fluctuate unpredictably around their true values) or systematic (where measurements fluctuate predictably around their true values).^1^^,^^2^ Our focus is on random error in which an underlying variable of interest is measured with random variation (either due to true random fluctuations or imprecision of measurement).

Regression dilution bias (RDB), also known as attenuation by errors,^3^ refers to attenuation of an estimate between any covariable (i.e. exposure, confounder or mediator) and an outcome towards the null due to random measurement error in that covariable.^3^ Epidemiological studies often comment, in the discussion, on how findings might be underestimated because of RDB in an exposure without considering random measurement error of confounders that may impact exposure estimates in unanticipated ways.^1^ It has also been noted that where random measurement error is controlled for, this is often only done for exposure–outcome associations without making any attempt to control for random error in confounders.^3^^,^^4^ Whilst each confounder–outcome association would be biased towards the null in the presence of random error in that confounder, the impact of this random error on the main exposure–outcome effect would depend on the (unbiased) direction and size of effect of the confounders on the exposure and outcome.^1^ Thus RDB in confounders, as well as in the exposure, could alter the exposure–outcome effect estimates in either direction.

Some studies invite a subset of participants back for repeat assessments with the express purpose of providing researchers with data that could be used to correct for random measurement error, but these data seem to be rarely used. The Avon Longitudinal Study of Parents and Children (ALSPAC) repeated all 13 clinic assessments of parents and children undertaken over the last ∼30 years^5^^,^^6^ in a subset of 3% of participants and UK Biobank (UKB) did the same in up to ∼49 000 participants (∼10%) to enable assessment of potential random measurement error.^7^ We have only identified three papers in ALSPAC^8–10^ and four papers in UKB^11–14^ that have utilized repeat measures, in all cases to correct for RDB in exposure–outcome associations only. Thus, even where data are available to address random measurement error, they are rarely used, and the research resources and participant time and effort to collect these data are largely wasted.

The aim of this paper was to assess random measurement error between repeat measures of all continuous variables in UKB and explore approaches to mitigate its impact when estimating exposure–outcome associations. First, we assessed whether random or systematic error was likely present using intraclass correlation coefficients (ICCs)^15^ and accuracy coefficients.^16^^,^^17^ Second, we used an illustrative example to exemplify how to account for random error in exposure and confounders using ICCs and regression calibration. We provide ICCs and adaptable code demonstrating how to adjust for RDB in exposure– and confounder–outcome associations so that other researchers can assess and account for random measurement error in their own analyses. We have used UKB data as the scope for use was large; at the end of 2021, there were 25 000 registered researchers, with >4600 publications, with the number increasing exponentially from 120 in 2016 to 1700 in 2021 (Personal communication; Naomi Allen, Chief Scientist UK Biobank, April 2022).

## Methods

### Study data

UKB is a large prospective cohort study of >500 000 adults with a target age range of 40–69 years at recruitment (5.5% response of those invited) open to researchers for health-related research.^7^ The initial assessment, between 2006 and 2010, included physical measurements, participant-completed responses to computer questionnaires, as well as laboratory analysis of blood and urine samples. Around 20 000 participants were invited back for a repeat visit in 2012–13 when most measurements and sample assays were repeated. Some additional repeated information was collected via online questionnaires at other times for ≤49 000 participants, e.g. the 24-h diet survey (here we use two repeat measures from comparable online surveys and not the measure from the baseline visit). Imaging data were collected during visits starting in 2014 and repeated from 2019. Imaging data collection is ongoing; here we present data for the first 4498 participants with repeat measures.

**Figure 1:**
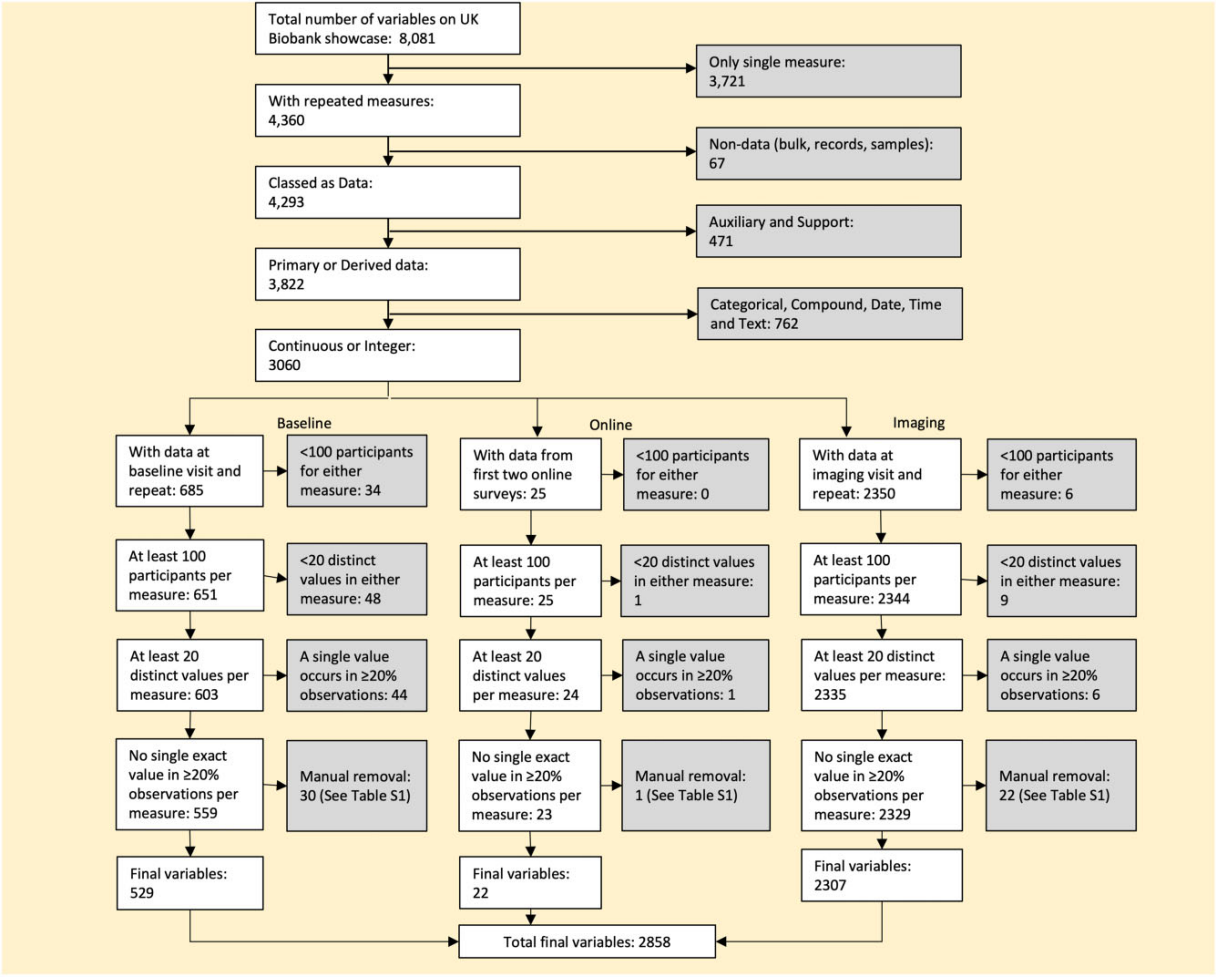
Data flow chart

From the full list of variables available on UKB showcase^18^ we excluded those without repeat measures, those that were not feasible covariables in regression analyses (e.g. data processing information) and those that were not continuous measures (or integer measures that could be treated as continuous, having >20 distinct values). We further excluded variables where >20% of participants had the same value, and those where <100 participants had repeated measurements (Figure 1 and Supplementary Table S1, available as Supplementary data at *IJE* online).

Information on how we dealt with values expressed as e.g. ≤1 mile and those with multiple readings at a visit is provided in Supplementary Text S1 and Supplementary Tables S2 and S3 (available as Supplementary data at *IJE* online).

### Statistical analyses

#### Assessing error in repeat measures

We calculated the ICC for each variable, which shows the level of agreement between two sets of measurements. In this test–retest situation, i.e. repeated measures but separated by weeks or months and not necessarily under identical circumstances (e.g. the person doing the assessment may be different), the most appropriate method to assess the ICC uses a two-way mixed effects model^15^ (Supplementary Text S2, available as Supplementary data at *IJE* online). The ICC usually takes values of between 0 and 1, with higher ICC suggesting lower random measurement error.

We also calculated the accuracy coefficient, a measure of systematic bias, using Lin’s method.^16^^,^^17^ The accuracy coefficient is a measure of how close the line of best fit between two repeat measures is to a line of 45° through the origin (Supplementary Text S2, available as Supplementary data at *IJE* online). It is always >0 and ≤1. For values close to 1, the less likely there is to be systematic error as the average difference between the pairs of measurements is close to 0.

#### Correction for random measurement error

We focused on two commonly used methods: correction using the ICC^19^ and regression calibration.^20^ The ICC method can correct for RDB due to random error in the exposure but it is not possible to account for random error in confounders using this method. It involves first fitting a regression model to estimate the exposure–outcome association and then using the ICC statistic to correct this estimate and associated confidence interval.

The correction factor $\hat{\lambda }\;$is the reciprocal of the ICC.^15^ The uncorrected main effect estimate $\hat{\beta }$ e.g. difference in means, log odds, logit or log hazard, is multiplied by the correction factor to get the corrected estimate $\hat{\beta }$*. The confidence interval uses the following formula to take account of the uncertainty in the correction factor:


$$\text{95}\%\text{ CI for }\hat{\beta }*=\;\frac{f1\pm \sqrt{\left(f_{1}^{2}-f_{0}f_{2}\right)}}{f_{2}}$$

Where $f_{0}={\hat{\beta }}^{2}-1.96^{2}var(\hat{\beta })$


f1=β^λ^-1.962cov(β,^1/λ^)

f2=1λ^2−1.962var(1/λ^)       from Frost 200 019

The covariance $\mathrm{cov}(\hat{\beta ,}1/\hat{\lambda })$ is 0 if the sample of repeated measures is different to the main analysis sample. If the repeated sample is a small subset of the main sample, then the covariance will be close to 0 and the parameters can be treated as independent,^19^ which is what we assume in this paper. Formulae for the variances of $\hat{\lambda }$ and $1/\hat{\lambda }$ are in Supplementary Text S3 (available as Supplementary data at *IJE* online).

Regression calibration can be used to correct for bias due to random error in the exposure and confounders (or other covariables) together. It involves regressing the repeat measure of the exposure on the initial measure, including in the model the same confounders used in the main analysis model (first-stage model).^20^ Then the coefficients from this linear regression are used to predict fitted values of the exposure for the whole data set. These predicted values are used in the main model in place of the original values of the exposure (second-stage model). The effect estimate from this model is thus corrected for exposure–outcome RDB. To correct for random measurement error in both the confounders and exposure, a first-stage model is run for each covariable measured with error, adjusted for all other covariables (e.g. exposure and confounders). Predicted values from each of these first-stage models are then used in the second-stage model. To account for uncertainty in both stages we recommend bootstrapping the whole process to obtain corrected confidence intervals.^20^

Both the above methods can provide unbiased estimates of association with linear regression and approximately unbiased estimates for non-linear Cox proportional hazards and logistic regression.^20^^,^^21^

#### Illustrative example

To illustrate these methods, and the potential impact of random measurement error on associations, we estimated associations of red blood cell distribution width (RDW), C-reactive protein (CRP) and 25-hydroxyvitamin D [25(OH)D], with all-cause mortality. These three were chosen because they had evidence of modest to strong random measurement error and because they have all been shown to be associated with several chronic conditions that predispose to premature mortality and/or mortality.^22–30^ Of these, previous studies using Mendelian randomization suggest a causal effect of higher CRP on schizophrenia, though not cardiovascular disease, cancers or several other chronic diseases, and of 25(OH)D on all-cause and cancer mortality.^31–34^ Furthermore, all may be accurate predictors for premature mortality and correction for random error is important whether the multivariable regression analysis is exploring causality or just association.

Confounders were selected a priori on the basis of being known or plausible determinants of both the exposure and mortality outcome. These were sex, age, ethnicity, Townsend home neighbourhood area deprivation score, lifetime smoking pack-years, alcohol consumption and body mass index (BMI). Full details of how these were measured and categorized are provided in Supplementary Text S4 (available as Supplementary data at *IJE* online). Four of these confounders—age, area deprivation, smoking and BMI—were continuous. Of these, repeat measures were not available for area deprivation. Difference in age between the two repeat measurements accurately reflected the participants ageing and so was not considered a potential source of random measurement error. Thus, we corrected for potential bias due to random error in smoking and BMI. It is possible that a non-linear format is more appropriate for some confounders (e.g. both higher and lower BMI may increase mortality), which is not within the scope of this paper.

We fitted Cox proportional hazards models using date of baseline assessment and date of death or end of follow-up (28 February 2021) for those who survived to calculate years of follow-up. Mortality data were available in the UK Biobank for all participants via linkage to UK death registries.

For each outcome we present confounder-adjusted results with: (i) no correction for random measurement error; (ii) correction for RDB due to exposure random error using ICC; (iii) correction for RDB due to exposure random error using regression calibration; and (iv) correction for random error in both exposure and (continuously measured) confounders using regression calibration. Bootstrapping was used over the whole regression calibration process (first-stage linear regression followed by second-stage Cox proportional hazards) with 10 000 replicates to calculate confidence intervals.

All code is available at https://github.com/MRCIEU/measurement_error_adjustment/blob/master/README.md. Git tag v0.1 corresponds to the version presented here. All analysis was completed using Stata version 16.^35^

## Results

### Assessing error in repeat measures

Of the 8081 available UKB variables we were able to provide correction factors for 2858. Split into three groups, these included 529 baseline visit variables, 22 online diet questionnaire variables and 2307 medical imaging visit variables. Most excluded variables were due to not having a repeat measure (*n* = 3721) or not being continuously measured (*n* = 762) (Figure 1).

Supplementary Tables S4–S6 and Supplementary Figures S1–S10 (available as Supplementary data at *IJE* online) provide metadata on the variables with repeat measures, including time between repeat measures, available sample size, ICC, accuracy coefficient and correction factor statistics. The median time between the repeated visits in baseline measurements varied between 34 and 56 months. All the online diet by 24-h recall variables had a median of 4 months between visits and all the imaging visit variables had a median of 27 months between visits. The sample size of repeated measures across all variables varied from 135 to 45 836.

**Figure 2:**
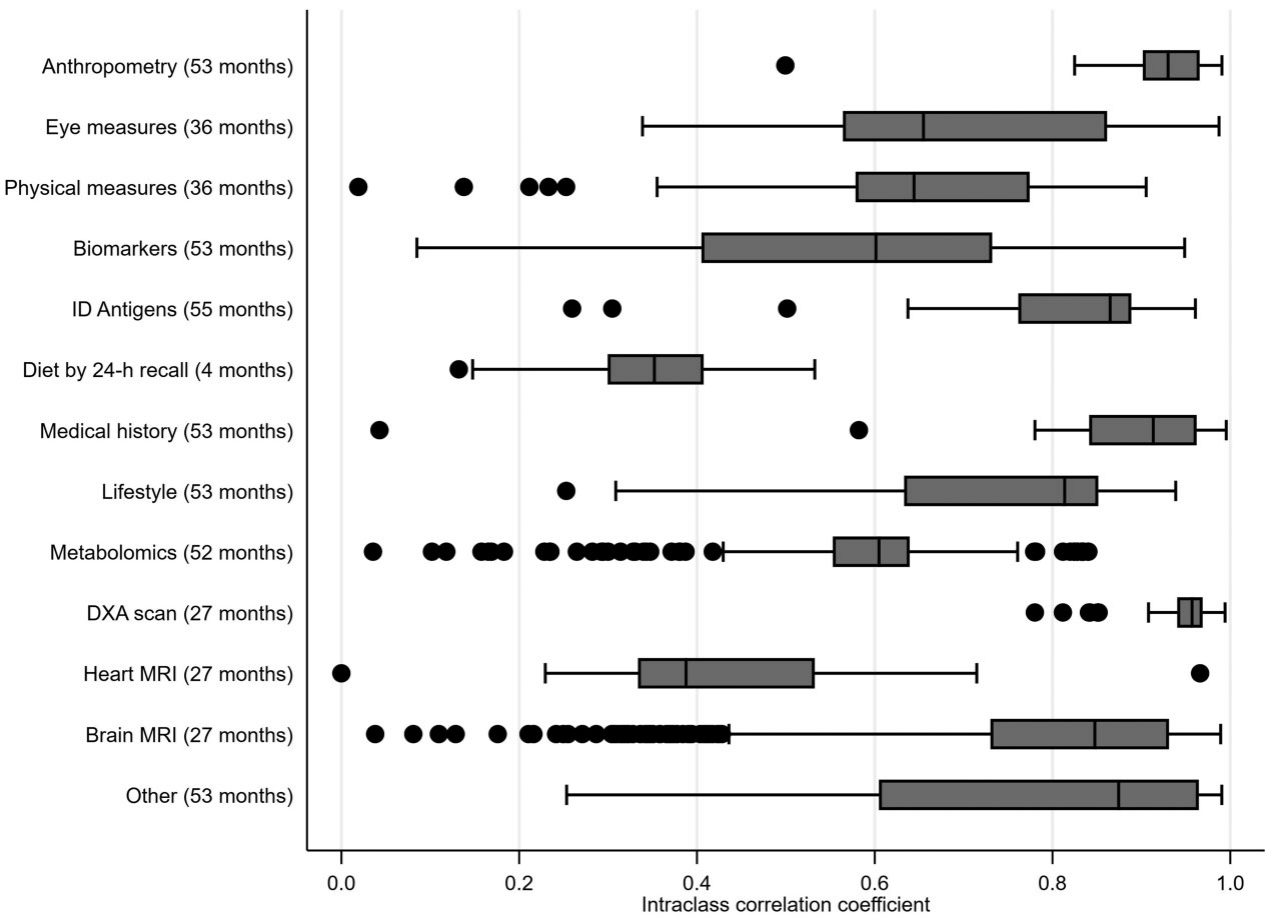
Box and whisker plot of intraclass correlation coefficients measuring overall agreement between repeated measurements by variable category. The months in brackets after each category name are the median time between the first and second measures. For each category the vertical line inside the horizontal box reflects the median intraclass correlation coefficient for that category; the box denotes the interquartile range (IQR); the vertical lines at the end of the protruding horizontal lines from each box reflect the adjacent values (values within 1.5 times the IQR) and the dots denote separate points more extreme than the adjacent values. ID, infectious disease; DXA, dual-energy X-ray absorptiometry; MRI, magnetic resonance imaging

**Figure 3:**
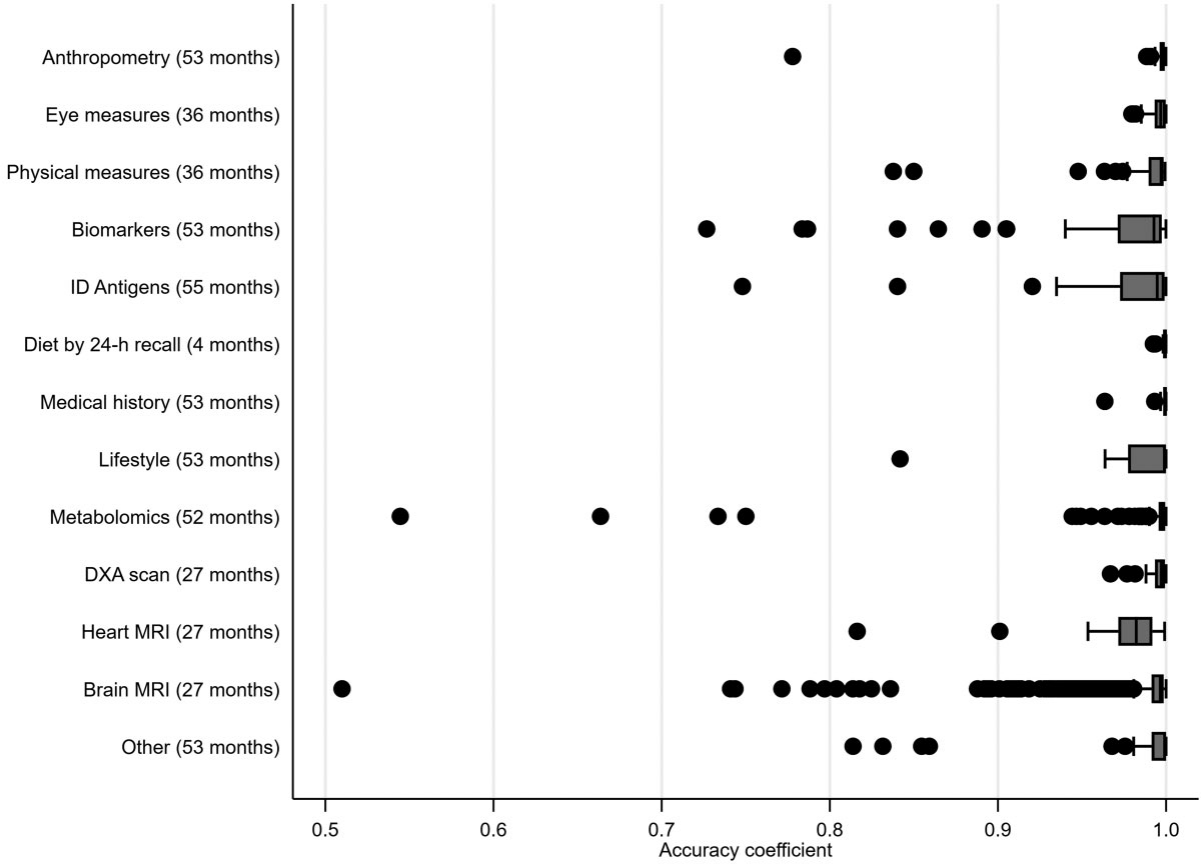
Box and whisker plot of accuracy coefficients measuring systematic difference between repeated measurements by variable category. The months in brackets after each category name are the median time between the first and second measures. For each category the vertical line inside the horizontal box reflects the median accuracy coefficient for that category; the box denotes the interquartile range (IQR); the vertical lines at the end of the protruding horizontal lines from each box reflect the adjacent values (values within 1.5 times the IQR) and the dots denote separate points more extreme than the adjacent values. ID, infectious disease; DXA, dual-energy X-ray absorptiometry; MRI, magnetic resonance imaging

**Figure 4:**
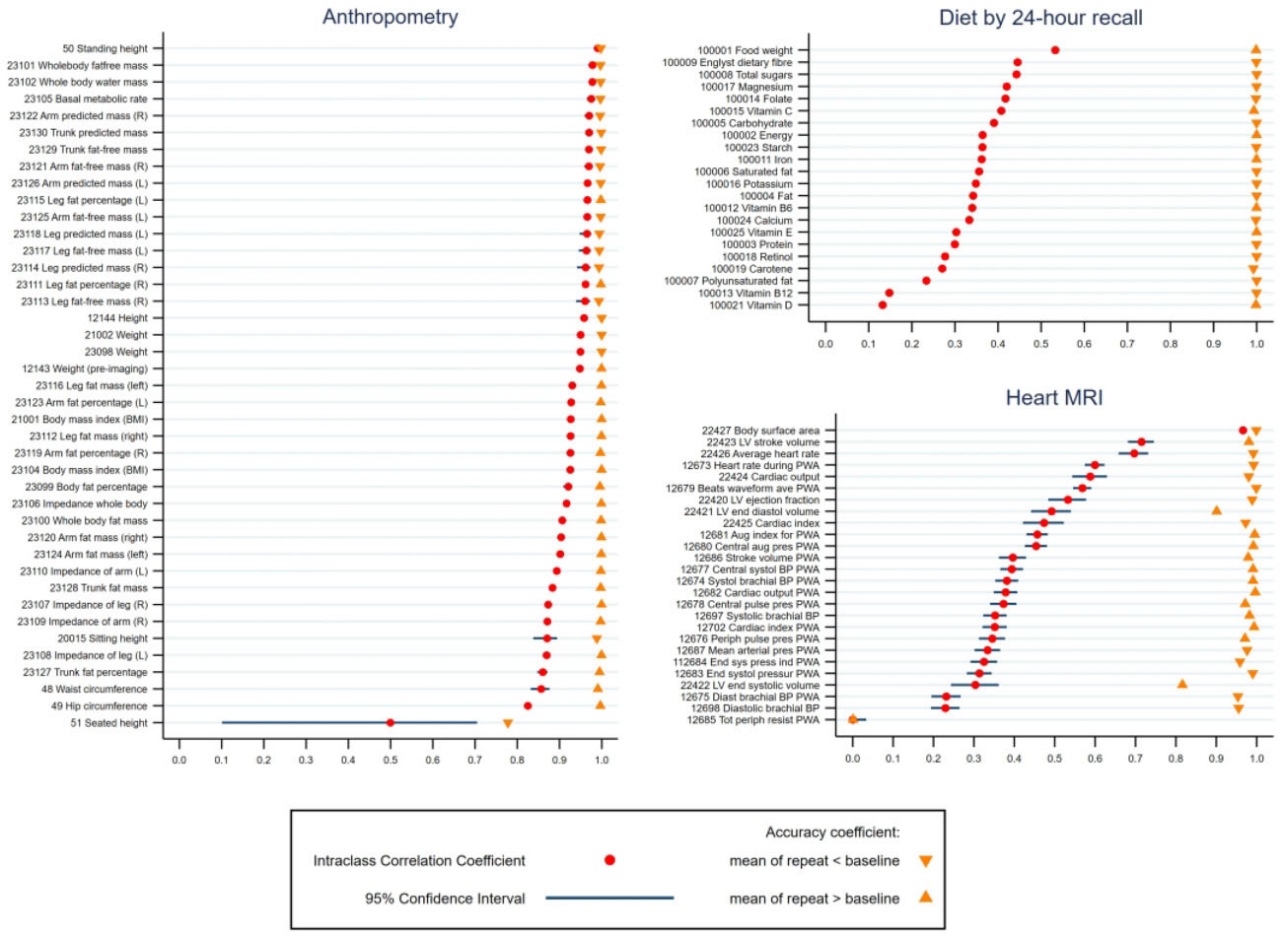
Intraclass correlation and accuracy coefficients for variables in anthropometry, diet by 24-h recall and heart magnetic resonance imaging. Results are the intraclass and accuracy coefficients with 95% CI for each variable in the three categories. Where the coefficients are very precisely estimated the 95% CIs are not clearly visible. Coefficient values and their 95% CIs for all 2858 variables (i.e. those in this figure and all of those in other categories not shown in this figure) are provided in Supplementary Tables S4–S6 (available as Supplementary data at *IJE* online). There are multiple variables for height and weight: 50 Standing height—measured as part of the baseline assessment; 12 144 Height—measured in the subgroup of participants attending the detailed imaging assessments as it is required to calibrate the DXA scans; 21 001 Weight—a derived variable amalgamating multiple methods of measurement at the assessment centre visit; 23 098 Weight—taken during impedance measurement; 12 143 Weight (pre-imaging)—measured in the subgroup of participants attending the detailed imaging assessments as it is required to calibrate the DXA scans. DXA, dual-energy X-ray absorptiometry

Overall, the online diet by 24-h recall variables had the lowest ICCs [median = 0.35, interquartile range (IQR)=(0.30, 0.40)], baseline visit, mostly self-completed questionnaire variables in between [median = 0.64, IQR = (0.57,0.82)] and imaging visit variables the highest [median = 0.85, IQR=(0.73, 0.94)]. Across all variables, 7% had ICCs of <0.50, 28% had ICCs of between 0.50 and 0.74, 29% had ICCs of between 0.75 and 0.89, and 36% had ICCs of ≥0.90. By variable category, the highest ICCs were seen in anthropometric, medical history and dual-energy X-ray absorptiometry (DXA) scan variables and the lowest in 24-h recall dietary measures and heart MRI (Figure 2). Most variables had accuracy coefficients of >0.95 (Figure 3 and Supplementary Tables S4–S6, available as Supplementary data at *IJE* online) indicating generally low systematic error, though the amount of systematic error differed by variable category [Figure 4 shows illustrative ICCs and accuracy coefficients together for anthropometric, diet by 24-h recall and heart MRI; with similar figures for individual variable categories in Supplementary Figures S1–S10 (available as Supplementary data at *IJE* online), except metabolomics, DXA scan and brain MRI due to number of variables]. As an example, standing height had a high ICC and high accuracy, whereas seated height had a high ICC and low accuracy (Figure 3). This was due to different height boxes being used to sit on, with more taller boxes used at the second visit than the first. This might not be easily detected by researchers without checking the repeat measurements and then the UKB documents to understand this.

### Correction for regression dilution bias—an illustrative example

The ICC for RDW suggested moderate random error [ICC = 0.52, 95% CI=(0.51, 0.53)], for CRP substantial random error [0.29 (0.27, 0.30)] and for 25(OH)D moderate random error [0.55 (0.54, 0.56)]. Little random error was found for BMI [0.93 (0.92, 0.93)] and pack-years of smoking [0.85 (0.84, 0.86)]. The accuracy coefficients for all five were ≥0.99 indicating little systematic error (Table 1 and Supplementary Table S4, available as Supplementary data at IJE online).

**Table 1:** Measures of random and systematic error for red blood cell distribution width, C-reactive protein, 25 hydroxyvitamin D, body mass index and pack-years of smoking for illustrative examples

UK Biobank field number	Description	Sample size	Accuracy coefficient^a^	Intraclass correlation coefficient^b^ (95% CI)	Correction factor^c^, $\hat{\lambda }$	Var($\hat{\lambda }$)	Var(1/$\hat{\lambda }$)
30070	Red blood cell distribution width	18 379	1.00	0.52 (0.51, 0.53)	1.9356	0.000410	0.000029
30710	C-reactive protein	16 549	1.00	0.29 (0.27, 0.30)	3.4987	0.007635	0.000051
30890	25 hydroxyvitamin D	15 435	0.99	0.55 (0.54, 0.56)	1.8091	0.000335	0.000031
21001	Body mass index	20 257	1.00	0.93 (0.92, 0.93)	1.0794	0.000001	0.000001
20161	Pack-years of smoking	4744	1.00	0.85 (0.84, 0.86)	1.1745	0.000030	0.000016

a Accuracy coefficient is a measure of systematic error between two measures.

b Intraclass correlation coefficient is a measure of overall agreement between two measures.

c Correction factor ($\hat{\lambda }$) is the reciprocal of the intraclass correlation coefficient.

For the three exposures there were between 3.57 and 3.81 million person-years of follow-up, which is an average of 11.8 years per participant. For RDW there were 23 509 deaths, giving an all-cause mortality rate of 6.17 per 1000 person-years [95% CI = (6.09, 6.24)]; comparative rates for CRP and 25(OH)D were 6.16 [95% CI = (6.08, 6.24)] and 6.14 [95% CI = (6.06, 6.22)], respectively.

In confounder-adjusted analyses, higher RDW and levels of CRP were associated with higher risk of all-cause mortality, and higher concentration of 25(OH)D was associated with lower risk of all-cause mortality (Table 2). When these analyses were corrected for random error in the main exposure (using either ICC correction or regression calibration) associations moved further from the null. With further correction for random error in the smoking and BMI confounders there was little further change (Table 2).

**Table 2:** Cox proportional hazard results for red blood cell distribution width (RDW), C-reactive protein (CRP) and 25 hydroxyvitamin D [25(OH)D] associations with all-cause mortality, adjusted for sex, age, ethnicity, body mass index (BMI), smoking pack-years, drinking and deprivation index

Model	All-cause mortality hazard ratio per 1% increase in the RDW (95% CI) *N* = 324 467	All-cause mortality hazard ratio per 1-mg/L increase in CRP (95% CI) *N* = 317 917	All-cause mortality hazard ratio per 1-nmol/L increase in 25(OH)D (95% CI) *N* = 303 858
(i) Uncorrected Cox proportional hazard	1.203 (1.194, 1.212)	1.029 (1.028, 1.031)	0.993 (0.992, 0.994)
(ii) Corrected for regression dilution in main exposure using intraclass correlation coefficient	1.430 (1.407, 1.453)	1.107 (1.099, 1.116)	0.988 (0.986, 0.989)
(iii) Corrected for regression dilution in main exposure using regression calibration	1.482 (1.398, 1.567)^a^	1.119 (1.095, 1.143)^a^	0.988 (0.987, 0.990)^a^
(iv) Corrected for regression dilution in main exposure and both BMI and smoking pack-years (confounders) using regression calibration	1.480 (1.394, 1.565)^a^	1.118 (1.095, 1.142)^a^	0.989 (0.987, 0.990)^a^

a Bootstrapped 95% CIs with 10 000 replications.

## Discussion

In this paper we provide summary ICCs and accuracy coefficient data for 2858 UKB continuously measured repeatedly assessed variables. We also provide code that researchers can adapt to explore the impact of random measurement error in exposures and confounders in future UKB studies. This is important as a subgroup of participants had a repeat assessment at cost to the funders and participants in order to support assessment and correction for random measurement error, but to date this has been done for just a tiny fraction of the UKB studies. The provided analysis code could also be useful for researchers wanting to explore the impact of random measurement error in other studies with repeat measures.

In our illustrative examples key confounders had high ICCs and hence correction of confounders for bias due to random measurement error did not substantially change the exposure-corrected results. This might not be the case in other studies with different confounders as well as different populations and different ways of measuring. There will be examples in which there will be considerable confounder random error for a given research aim and in those cases it will be important to correct for this.

### Assumptions of correcting for random measurement error including RDB

The magnitude of the ICC may reflect random error and/or systematic changes (e.g. change in measurement method, age-related change). Here we used the accuracy coefficient as a proxy to identify possible systematic changes with the assumption that non-random error would show up as a difference in the mean values of the two measurements. However, this will not always be the case and some systematic changes will not be identified this way (e.g. if one group consistently increased and another consistently decreased this could result in the same mean value). As such, possible reasons for systematic difference need to be considered for each variable of interest.

In this study the difference in time between measurements varied from a median of 4 to 53 months for different categories of variable. We might anticipate that greater time between variables would increase the likelihood of true (not random) differences. For example, as adults age they on average eat less and different types of food,^36^ and these differences will likely increase with time. The two measures with the longest time between repeats were smoking variables and measurements of antigens to different infectious diseases, each with average differences of 53 months. Over that time differences in pack-years of smoking could reflect true changes in behaviour and differences in antigen levels could reflect chronic infection, rather than random variation.

However, our findings do not fully support this. For example, the shortest time between measures (median 4 months) was for the dietary recall variables but this category had one of the lowest median ICCs. By contrast, the medical history category with a high median ICC had one of the longest time differences (median 53 months), which might reflect true changes in health over this time. Heart MRI measures had a low median ICC but by contrast brain MRI measures had a high median ICC despite the median time between measurements being the same (27 months) and for individuals the time difference will have been identical as these two measures are taken in the scanning clinic at the same visit for a given participant. Thus, other factors in addition to time differences might influence the ICC.

A further assumption is that there is some true underlying or average value of the exposure of interest that is either measured with error, fluctuates regularly or both. If a specific measurement at a particular time point is the target variable, then adjusting for RDB would not be appropriate. Dietary measures for example are based on 24-h recall of foods eaten and if a person’s food intake does vary from day to day then their report might be accurate. However, derived variables from this 24-h recall in UKB and other studies are used to reflect the underlying average amount of macro and micronutrients consumed each day and, as our results show, this has considerable measurement error. Additionally, in our illustrative example we were interested in the effect of the underlying average values of RDW, CRP and 25(OH)D, not the values on a particular day that may fluctuate due to acute infection, diet or season. As exposure of the skin to ultraviolet-B is the major source of 25(OH)D, its levels vary by season, which is likely a major contributor to the ICC for this variable (e.g. if someone had blood taken for the first measure in winter and the second in summer, we would expect notable differences). This is random variation and, as we are interested in the effect of the underlying average concentration, it is important to correct for RDB for 25(OH)D and other variables with seasonal variability when they are exposures or confounders.

Another assumption when correcting for bias due to random measurement error in covariables is that the random error (identified using repeated measures of the covariable) is an accurate estimate of the error. If it is not, the estimate may be differential with respect to the outcome (i.e. the estimated random error in the covariable is related to the observed values of the outcome when by definition true random error would not be). If this assumption does not hold then the association could be biased in either direction and results of adjustment could be invalid.

Lastly, in correcting for random error in an exposure and some confounders there is an implicit assumption that, for the corrected result to be an unbiased estimate, no other (e.g. binary or categorical) confounders are also subject to random measurement error and no other bias exists (e.g. residual confounding, selection bias, systematic measurement error) otherwise results may still be biased in either direction. If bias from random measurement error is in the opposite direction to bias from another source then adjusting for it may cause estimates to be further from the true value.

These assumptions should be carefully considered to determine the appropriateness of correcting for random error in exposures and confounders; if it is unclear whether the assumptions have been met, results adjusted for measurement error in covariables might be best considered as sensitivity analyses.

### Study strengths and limitations

The main strength of this paper lies in the large sample size and breadth of variables available in UKB. This has allowed us to explore the extent that random error varies across different categories of variables. The fact that UKB is widely used means there is the potential for this paper to help increase the number of studies correcting for random error in exposures and confounders, and the methods can also be used for other studies where repeat measures are available.

One limitation is the length of time between repeat measurements in UKB, which was greatest and most variable for the baseline assessment repeats (median 53 months) with consistent and shorter gaps for the online diet and image variables. With longer periods between measures, differences may be more likely to reflect true systematic differences as discussed in the assumptions above.

Random misclassification in categorical covariables can cause bias in the association of interest but current methods for dealing with bias due to random measurement error cannot address this (unless there are a large number of ordinal categories that can be treated as continuous). Such measurement error, along with other forms of potential bias (such as non-random error in exposure, confounders and outcome), should always be considered when interpreting results.

### Conclusions and recommendations

Greater consideration should be given to exploring bias due to random error in exposures and confounders. Investigators setting up prospective studies should be encouraged to provide repeat measures in a subsample for this purpose. If repeat measurements are available then they should be used to investigate and potentially account for random error in exposures and confounders.

It is important to explore random error in all covariables (where possible) before using an adjustment. Adjusting for RDB in the exposure only will never result in a weaker association being found. However, if there is also random error in the confounders then the estimates could be biased in either direction. Where repeat measures are not available in a study of interest, it may be possible to use a correction factor from another study (such as the ones provided here from UKB) to correct for exposure–outcome RDB correction but this would not allow correction for random error in confounders. This assumes that the variable was measured in the same way in the two studies.

For transparency we recommend presenting all results in analyses, as in our illustrative example, including the ICCs and accuracy coefficients for exposures and confounders (where possible), along with three sets of results that account for: (i) no measurement error in covariables, (ii) measurement error in exposure and (iii) measurement error in exposure and confounders.

## Ethics approval

UKB received ethical approval from the UK National Health Service’s National Research Ethics Service (ref 11/NW/0382).

## Supplementary Material

dyad082_Supplementary_Data

## Data Availability

The data are available upon request to UKB.
